# Strained Mechanical and Fracture Analyses of Armchair-Chiral-Zigzag-Based Carbon Nanotubes Using Molecular Dynamics Simulations

**DOI:** 10.1021/acsomega.4c04323

**Published:** 2024-11-28

**Authors:** Ama tul Zahra, Jamoliddin Razzokov, Muhammad Kashif, Umedjon Khalilov, Haipeng Li, Kun Luo, Aamir Shahzad, Guogang Ren, G. Reza Vakili-Nezhaad

**Affiliations:** † Modeling and Simulation Laboratory, Department of Physics, Government College University Faisalabad (GCUF), Allama Iqbal Road, Faisalabad 38040, Pakistan; ‡ Institute of Fundamental and Applied Research, National Research University TIIAME, Kori Niyoziy 39, Tashkent 100000, Uzbekistan; § Department of Information Technologies, Tashkent International University of Education, Imom Bukhoriy 6, Tashkent 100207, Uzbekistan; ∥ Department of Biomedical Engineering, Tashkent State Technical University, Tashkent 100095, Uzbekistan; ⊥ Arifov Institute of Ion-Plasma and Laser Technologies, Academy of Sciences of the Republic of Uzbekistan, Tashkent 100125, Uzbekistan; # New Uzbekistan University, Tashkent 100007, Uzbekistan; ¶ School of Materials Science and Physics, China University of Mining and Technology, Xuzhou City 221116, China; ∇ School of Materials Science and Engineering, Changzhou University, Changzhou 213164, P R China; ○ School of Physics, Engineering and Computer Sciences, University of Hertfordshire, Hatfield AL10 9AB, U.K.; ⧫ Petroleum and Chemical Engineering Department, College of Engineering, Sultan Qaboos University, Muscat 123, Oman

## Abstract

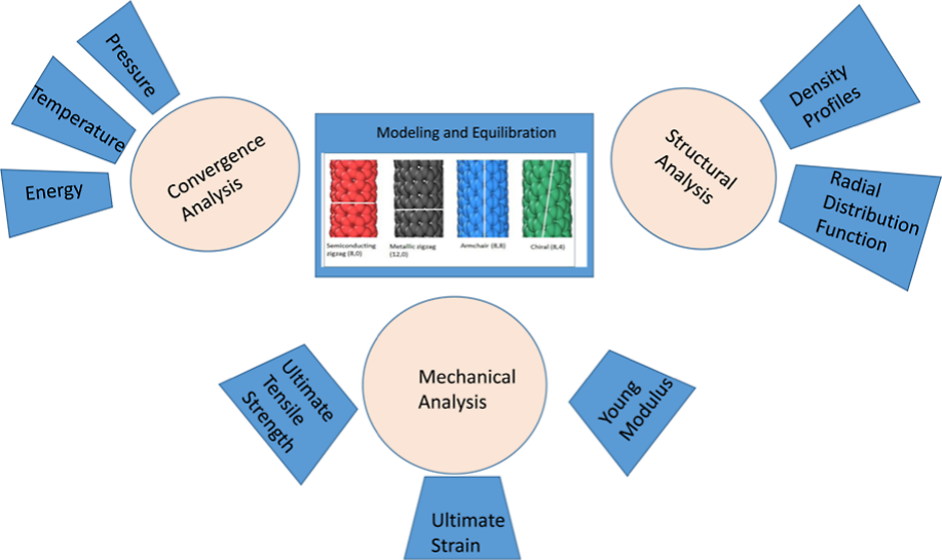

Carbon nanotubes (CNTs) have emerged as one of the most capable and interesting materials in recent decades and have extraordinary mechanical properties (MPs) and resourceful applications in bioengineering and medicine. Equilibrium molecular dynamics simulations have been performed to investigate the structural and MPs of armchair, chiral, and semiconducting and metallic zigzag single-walled CNTs (SWCNTs) under varying temperature *T* (K) and compressive and tensile strains ±γ (%) with reactive bond-order potential. New results elaborate on the buckling and deformation mechanisms of the SWCNTs through deep analyses of density profiles, radial distribution functions, structural visualizations, and stress–strain interactions. Density profile and structural visualizations of SWCNTs provide the understanding of atomic arrangements and structural changes under varying ±γ (%) strains. The structure of SWCNT configurations is changed at varying ±γ (%) and *T* (K) and radial distribution functions present the appropriate peaks for buckling and deformation states. It has been shown that the mechanical responses of different chirality of the SWCNTs clarify the variations in tensile strength in terms of *T* (K) and chirality. Stress–strain analyses reveal that the metallic zigzag and armchair SWCNTs have superior tensile strength as compared to chiral ones, having the lowest tensile strength. Simulation results show that yield strength, ultimate tensile strength, and Young’s modulus are higher for metallic zigzag and armchair SWCNTs at room *T* (K) and overall decrease with increasing *T* (K). However, the ultimate strain of semiconducting zigzag and armchair SWCNTs is higher as compared to other configurations, and it reflects the MPs of SWCNTs have to shed light on potential applications in nanotechnology and material sciences.

## 1 Introduction

Understanding of the mechanical characteristics of nanotubes under strains is of great interest for multidisciplinary applications such as sensors or smart materials. The exploration of mechanical properties (MPs) with their structural analysis is a challenging task for nano researchers, as it requires a comprehensive knowledge of their behaviors under different situations. The industrial performance of nanomaterials can be significantly enhanced due to their superior MPs.^1^ Ever-increasing demands for excellent performance and strong structures and uniaxial tensile strength of carbon nanotubes (CNTs) have been actively studied experimentally and computationally.^2^ The accurate numerical investigation of MPs of different configurations of single-walled CNTs (SWCNTs) is one of the main purposes in the area of nanomaterials and technology. Tensile strength of SWCNTs is a major feature used in the nanomaterial design process for various technologies and academic and advanced nanomaterial developments. Tensile strength (uniaxial) of different nanostructures including SWCNTs and graphene has been keenly investigated in the laboratory and by employing computational synthesis.^3^ The recent improvements illustrate the development in advanced nanomaterial engineering for the past one decade, and mostly such problems were chosen, for which the laboratory data can be estimated through the computer experiment and theoretical predictions and/or where the observation has an analytical application. The precise simulation data of MPs of SWCNTs is an essential task in the field of physics of the nanomaterial and nanotechnology, as different MPs and corresponding structural analyses are well explained by the deep understanding of experiments and validation of analytical equations. The CNTs have become a focus of deep research exploration due to their exceptional thermal, electrical, optical, and MPs imparting strength and stiffness, and it makes an ideal candidate for numerous nanomechanical systems, engineering, composites, and biomedical applications.^4−6^ The MPs of CNTs including flexibility, elasticity, exceptional strength and stiffness, and high thermal conductivity offer benefits to obtain very promising results in the area of aerospace, electronics, and transportation.^7^ Reliable information on MPs is also important for optimized nanodevice designs and applications in nanotechnology, and specifically, the provision of accurate data is required for various factors such as ultimate tensile strength (UTS), yield strength, and Young’s modulus, *Y* (Pa). A comprehensive atomistic knowledge of MPs of CNTs for a wide range of material parameters is an important task.^8^

This paper provides the review of past investigations performed in the last decades, which help to understand the update of the variation on the MPs of different nanostructures for the various ranges of nanotube parameters. Over the last two decades, a lot of research regarding the complicated mechanical characteristics of SWCNTs has been reported in order to reveal the potential applications for developing fibers or fillers in nanocomposites.^9^ Molecular dynamics (MD) simulation is used to examine the fracture behavior of zigzag, armchair, and chiral nanotubes to check dependency on chirality and separation energy.^10^ Radial deformability is one of the major elements that contributes to the buckling mode. An experimental investigation on two adjacent multiwalled CNTs (MWCNTs) reveals that the cylindrical symmetry of nanotubes becomes imperfect in anisotropic physical conditions.^11^ Young’s modulus *Y* (Pa) was reported to be in the range of 320–1470 GPa in the case of SWCNTs, while for MWCNTs, it varies from 0.27 to 0.95 TPa. Using density functional theory, the MPs of graphene were reported as the *Y* (Pa) to be 1.050 TPa and Poisson’s ratio to be 0.186. A slight change of chirality induces the variations in electrical properties of CNTs^12,13^ which can greatly influence the MPs. A theoretical study on axial stiffness, twisting, and rotation dynamics of SWCNTs explained a little dependence of *Y* (Pa) on the chirality and diameter of the nanotube and expected to be inflexible having *Y* (Pa) in the range of TPa.^14^ A temperature-related relation, which is proposed to examine elastic moduli of SWCNTs on different chiralities, showed that the temperature effects are least effective in zigzag CNTs as compared to others.^15^ The impact of high temperature on the compressive buckling of boron nitride nanotubes discloses the reduced structural stability and lower buckling loads and strains.^16^ Chirality and scale coefficient effects on the buckling load of zigzag double-walled CNTs (DWCNTs) are studied with axial compression using the nonlocal Timoshenko model.^17^ MD simulations of carbon nanostructures reveal that the zigzag structures have higher UTS than armchair ones and predicted *Y* (Pa) to be in the range of 1.31–1.83 TPa.^9^ WenXing et al.^18^ performed MD simulations and reported *Y* (Pa) = 0.9 TPa, showing little dependence on chirality and tube radius while Rafiee and Mahdavi^19^ used nondefected CNTs and found *Y* (Pa) to be in the range of 0.7–1 TPa, applying two potentials. The experimental study was conducted for MPs of suspended graphene sheets using an atomic force microscope and the *Y* (Pa) was extracted to be 0.5 TPa^20^ but using SWCNT ropes pulled by atomic force microscope tips *Y* (Pa) was reported to be in the range of 0.32–1.4 TPa.^21^ MWCNT composites with resin were analyzed by scanning electron microscopy micrograph and explained that the higher UTS is related to one with higher CNT content.^22^ The influence of various CNT types has been discussed regarding strength, while focusing the dependency on types and modification of cementitious composites.^23^ The use of machine learning in the prediction of MPs for cementitious materials with CNTs has also been explored.^24^ Different discrepancies regarding the information on MPs of the CNTs employing compressive and tensile strains arise from different factors like variations in configurations, input parameters, boundary conditions, investigation scheme, etc.^25^ The particular motivation of this work is the observation of atomic-scale variation of structural properties (stability analyses) through radial distribution function (RDF) and density profile tests with a variation of MPs in SWCNTs at high system temperatures and strain values.

The main purpose of this reported study is to investigate the effects of varying system temperatures and strains on the mechanical buckling/deformation analysis of the armchair-chiral-zigzag-based SWCNTs. The MPs (yield strength, UTS, and Young’s modulus) of armchair, chiral, and zigzag configurations are computed. The local nanostructures of armchair-chiral-zigzag-based SWCNTs are reported using deep visualization of RDF and density profiles. The mechanical buckling and deformation are captured through snapshots and measured by using UTS at varying system temperatures and strains.

## 2 Computational Methodology

**Figure 1:**
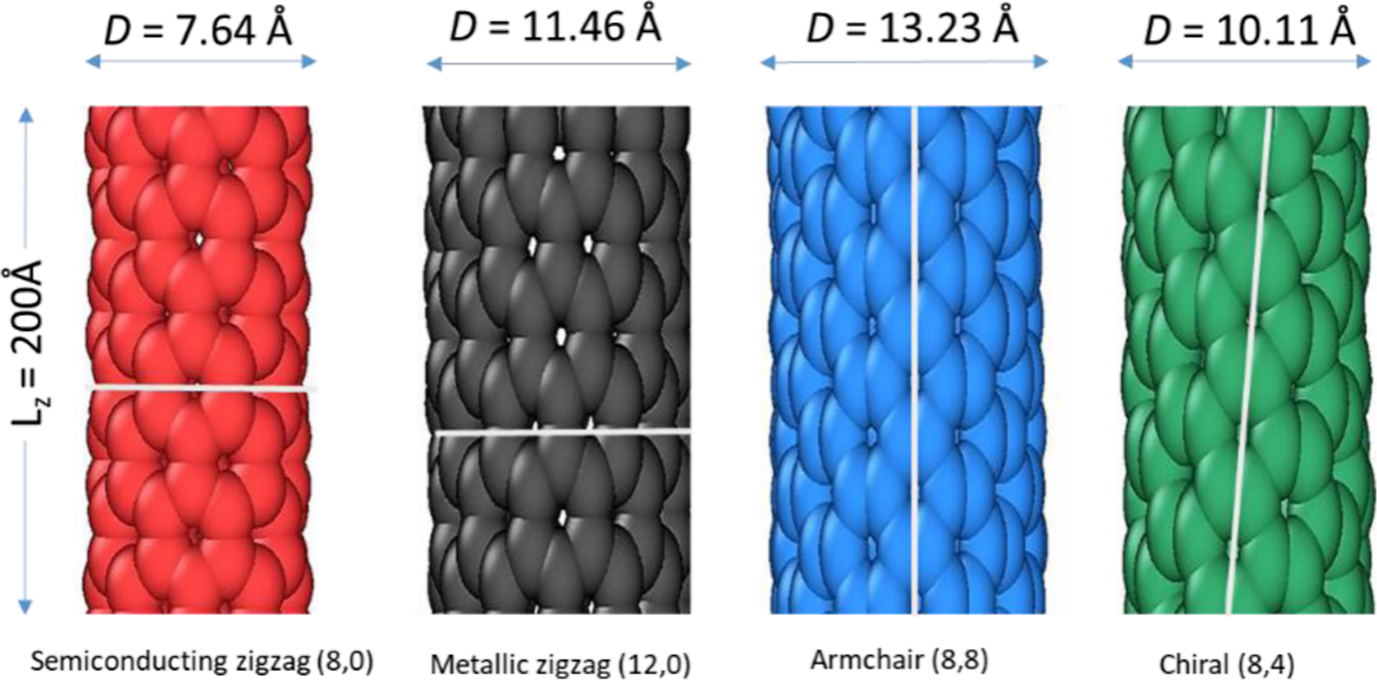
Visualizations of four initial configurations of semiconducting zigzag (8,0), metallic zigzag (12,0), armchair (8,8), and chiral (8,4) SWCNTs with *D* = 7.64, 11.46, 13.23, and 10.11 Å, respectively, and length *L* = 200 Å.

An analytical and descriptive research design for understanding the mechanical strength of different configurations of SWCNTs has been employed by using equilibrium molecular dynamics (EMD) simulations. Semiconducting zigzag (8,0), metallic zigzag (12,0), armchair (8,8), and chiral (8,4) SWCNTs of length, *L* (Å) = 200 Å are chosen to analyze the mechanical behavior having a nanotube radius of *r* = 3.820, 5.730, 6.616, and 5.053 Å, respectively. Each nanotube is designated a specific color, and their corresponding diameter is shown in Figure 1. MD simulations of semiconducting zigzag (red color), metallic zigzag (gray), armchair (blue), and chiral (green) SWCNTs are performed employing computer software LAMMPS (Large-scale Atomic/Molecular Massively Parallel Simulator) over a wide range of compressive −γ (%) and tensile +γ (%) strains.^5^

The current simulation study offers an atomic model system without friction and is accompanied by the application of periodic boundary conditions. All configurations of SWCNTs comprise sp^2^ hybridization in which one carbon atom is covalently bonded with three other carbon atoms. Initially, we performed an MD simulation in the *NPT* ensemble to bring the system to the desired pressure and temperature followed by the *NVT* ensemble under isothermal conditions at *T* (K) = 300, 500, and 700 K. Pressure *P* (Pa), temperature *T* (K), potential energy *E*_pot_ (kcal/mol), and total energy *E*_total_ (kcal/mol) are continuously monitored throughout the simulation run. The time step of d*t* = 200 ps is set to achieve the desired equilibration state for each configuration at specific *T* (K). Improved reactive bond-order (REBO) potential has been used to calculate the *E*_pot_ of covalent bonds and the related other interatomic interactions of SWCNTs. This potential can simulate bond breaking, chemical reactions involved in CNTs,^26^ and is given as
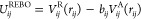
1where *V*^R^ and *V*^A^ represent the repulsive and attractive potentials, *b*_*ij*_ is the bond order term, and *r*_ij_ symbolizes the position defining the distance between *i*th and *j*th. Number density (ρ_*N*_) is a fundamental concept that delivers insights into the special distribution of particles within a material. It covers broad applications to understand material properties such as chemical reactions, phase transitions, and the performance of nanomaterials. It includes the interaction of factors such as thermal motion, phase changes, particle packing and their interactions, and is given as
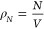
2where *N* represents the total number of particles (atoms/molecules) within a considering volume *V*. In our case, it is used to calculate the ρ_*N*_ along the length (*z* axis) of the respective SWCNTs. The main purpose of generating the density profile is to provide the structural information on SWCNTs. It helps in quantifying how closely/tightly particles are arranged within a specified volume. High ρ_*N*_ identifies a closely packed arrangement while low ρ_*N*_ indicates a more dispersed distribution of atoms. Visualizations of all configurations of SWCNTs have been captured through a version of the software on an OVITO computer at different time frames. Radial distribution function (RDF) or pair correlation function *g*(*r*) has been used to explore the structural characteristics of SWCNTs in three configurations including the aspects like the organization of atom-to-atom distance, and the level of arrangement or randomness present within the systems. The general expression of RDF is expressed as^5,26^
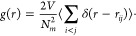
3

The function defines the local grouping formed around a reference atom and is related to the possibility of localizing an atom at a distance of *r*. The behavior of RDF peaks under varying strains (buckling or deformation) and temperatures is linked with the structural stability of the SWCNTs. It is used to analyze the structural properties of SWCNTs, and the orderness/disorderness in the system.^5,26^ RDF plots are made using visual MD (VMD) to analyze the structural characteristics of (8,0), (12,0), (8,8), and (8,4) SWCNTs when a combination of ±γ (%) are applied at *T* (K) = 300, 500, and 700 K.

To examine the effect of ±γ (%) on the structural and MPs of SWCNTs, all nanotubes are exposed to +γ = 1 to 40% and −γ = 0.1 to 10% at all given *T* (K). Applied strains depend on the deformation limit, which is different for each configuration at a specific *T* (K). Ultimate tensile strength and ultimate strain are determined at the given *T* (K) values using plotted stress–strain graphs. Temperature and chirality play an important role in analyzing MPs of SWCNTs with combinations of ±γ (%) and determining the *Y* (Pa) for each configuration. Young’s modulus is a material property that deals with stretching and deformation scale of materials^27^ and is defined as the ratio of tensile stress to strain.
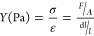
4where *F* is the applied force on unit area *A* while d*l* is the change in length and *l* is the original length.

## 3 Results and Discussion

Analysis of mechanical characteristics of SWCNTs at three different temperatures was obtained to better understand the structural behavior based on the buckling and deformation processes. Different values of ±γ (%) are used to identify the maximum buckling and breaking limit of (8,0), (12,0), (8,8), and (8,4) SWCNTs along with respective density profiles which vary with the temperature.

### 3.1 Convergence Analyses

**Figure 2:**
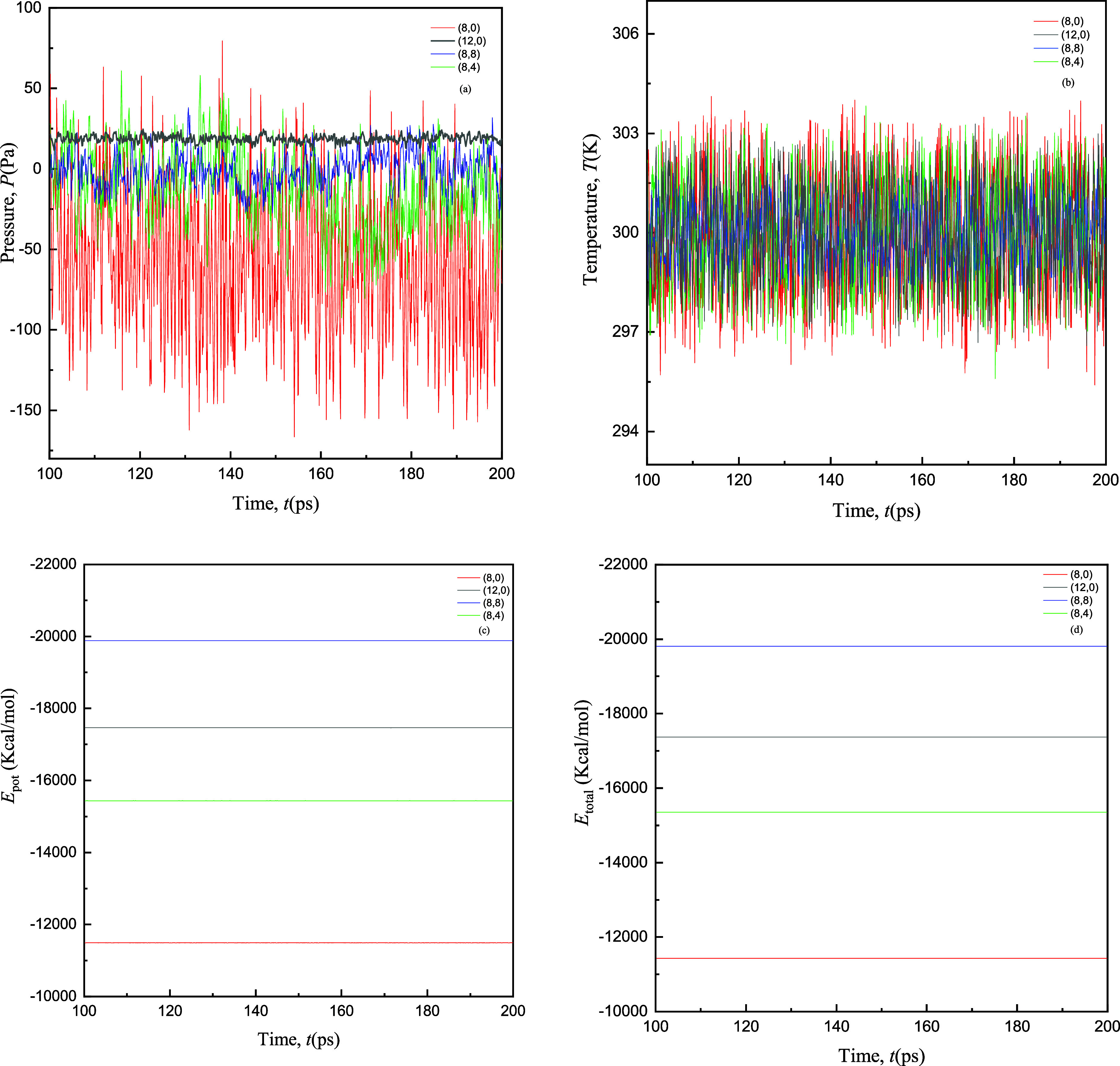
Convergence analyses of (a) pressure *P* (Pa), (b) system temperature *T* (ps), (c) potential energy *E*_pot_ (kcal/mol), and (d) total energy *E*_total_ (kcal/mol), respectively, for (8,0), (12,0), (8,8), and (8,4) using EMD simulations without strains (%) at *T* = 300 K.

In this subsection, convergence analysis is performed to examine the stability of the system when the system is unstrained. For the equilibration of CNT systems, an *NVT* ensemble is applied for monitoring different parameters to ensure that the system is in the equilibrium state. Figure 2 shows the convergence trends of pressure *P* (Pa) and temperature *T* (K) *w.r.t* time *t* (ps) that are observed throughout equilibration processes of the four configurations of SWCNTs at room temperature *T* (K) = 300 K. The first panel of Figure 2a signifies the convergence of *P* (Pa) as a function of *t* (ps), and it is linked to the structural stability of SWCNTs. It is examined that the *P* (Pa) of (12,0) metallic zigzag SWCNTs with large diameter converges more smoothly due to the inherent stability (metallic behavior) in contrast to the armchair (8,8), chiral (8,4), and semiconducting zigzag (8,0) SWCNTs. Furthermore, it is observed that (8,8) and (8,4) possess different structural arrangements leading to varying notches of fluctuations during convergence meanwhile (8,0) converge with comparatively high fluctuations. The earlier convergence can be due to the metallic nature and probably large diameter of (12,0) SWCNTs, leading to fast convergence and equilibration of the CNT system. It is well-known that the anisotropic nature of the CNTs’ bonding interactions and corresponding geometries contributes to distinctive behaviors.^28^

The second panel of Figure 2a represents the temperature analysis that is monitored throughout the simulation run for the same sets [(8,0), (12,0), (8,8), and (8,4)] of SWCNTs. Convergence of *T* (K) reveals the formation of a thermal equilibrium within SWCNTs. It is observed from the second panel that the *T* (K) of (12,0) and (8,8) configurations fluctuate with fewer notches of fluctuations, however, all configurations ultimately converge to stable states throughout the simulation run (*t* = 200 ps). The fluctuations around the equilibrium state within a range of ±3K are attributed to the exchange of energy between SWCNTs and the surrounding environment, as shown in Figure 2b. This temperature profile shows that the different configurations of SWCNTs throughout the simulation sustain a relatively stable thermal state.

Moreover, in the next two panels of Figure 2c,d, the corresponding *E*_pot_ (kcal/mol) and *E*_total_ (kcal/mol) analyses exhibit the convergence of SWCNTs as a manifestation of energy minimization processes at a specific value. Each SWCNT reaches a steady state when forces among atoms are balanced, resulting in minimized *E*_pot_. The equivalence of *E*_pot_ (kcal/mol) and *E*_total_ (kcal/mol) exhibits the absence of external work being performed on the system. When internal interactions dominate and no external factors influence the energy exchange then energy conservation prevails. Overall, convergence analyses observed in Figure 2 are the interplay of structural stability, thermal equilibration, and energy conservation within SWCNTs at *T* (K) = 300 K in the absence of strains.

### 3.2 Density Profiles and Structural Analyses

**Figure 3:**
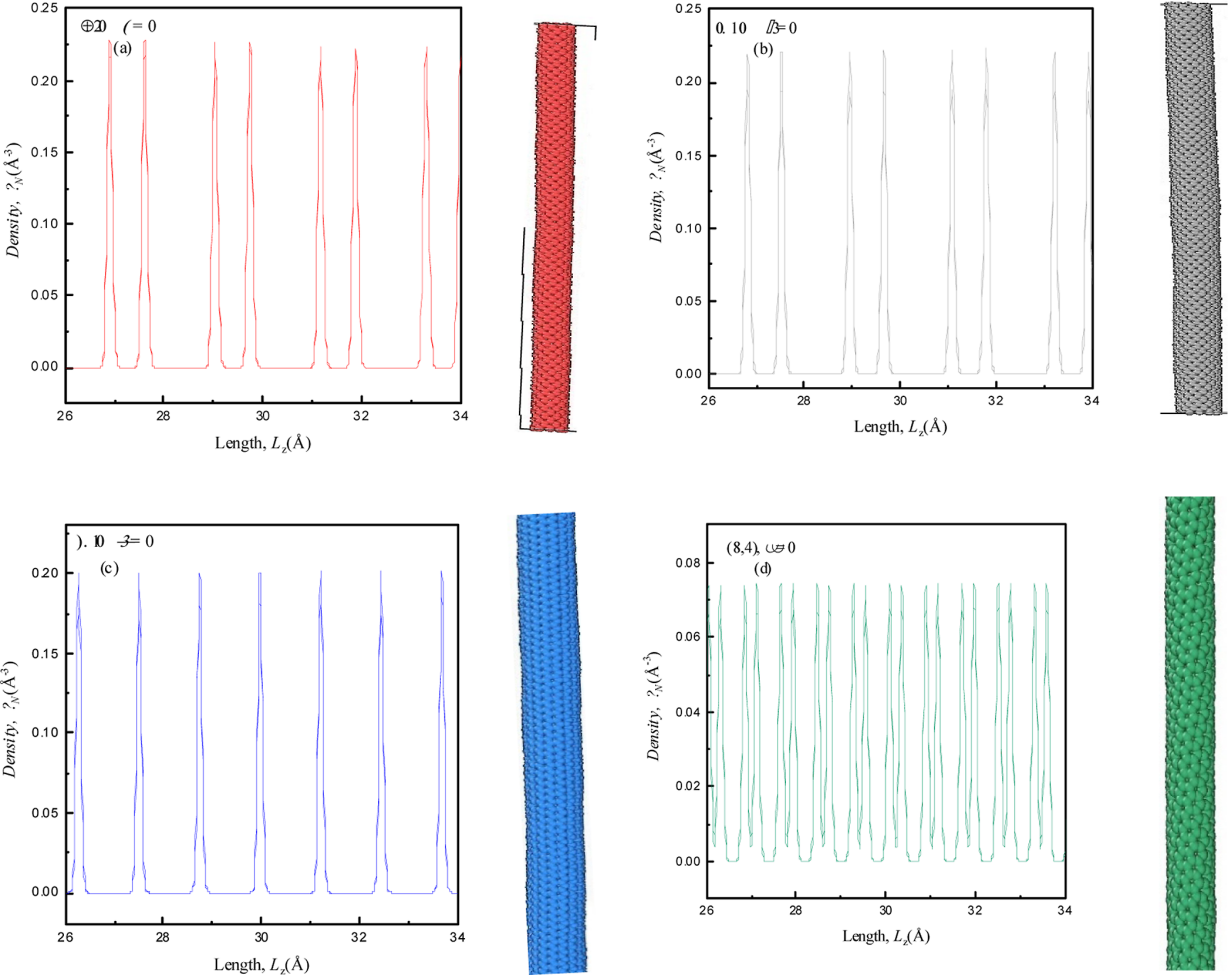
Density profiles obtained using the EMD simulation method along with corresponding structural visualizations (right side) without any strain (%) for (a) semiconducting zigzag (8,0), (b) metallic zigzag (12,0), (c) armchair (8,8), and (d) chiral (8,4) SWCNTs at *T* (K) = 300 K.

Chirality plays an essential role in determining the response of each SWCNT configuration toward applied tensile strains, resulting in distinct behaviors. Such findings help to develop material design by imposing these interconnections, with SWCNTs having specific material properties. Density profiles (ρ_*N*_) of the semiconducting and metallic zigzag, armchair, and chiral SWCNTs are examined in Figure 3 at *T* (K) = 300 K, in the absence of strain, focusing on a specific segment of length along the *z* axis, *L*_*z*_ (=26 to 34 Å), of the respective nanotubes to the detailed observation of the arrangement of peaks for each configuration. It should be mentioned here that a small segment of nanotube length *L*_*z*_ (=26 to 34 Å) is taken, for the magnification and clarity of bonds (and/or angles) in density profiles. The corresponding visualization (front views) of initial states of the zigzag semiconducting (8,0), zigzag metallic (12,0), armchair (8,8), and chiral (8,4) SWCNTs with diameters of *D* = 7.64, 11.46, 13.232, and 10.106 Å, respectively, are also shown in the right vertical side of respective panels at specific time frames.

Panels (a) and (b) illustrate that the density profiles of (8,0) and (12,0) zigzag SWCNTs show nearly the same patterns, and it may be due to the same chirality (zigzag). However, it is observed that the density distribution pattern of (8,8) is different from the density profile of (8,4) SWCNTs, showing that the SWCNTs are categorized based on chirality (nanotube’s indices and chiral angle), which significantly influences how atoms are tightly wrapped and bonded. Moreover, the diameter of the SWCNTs does contribute to its physical dimensions, but the chirality of nanotubes is mainly more involved because it can provide deep information regarding the complicated arrangement of carbon atoms. Consequently, the chirality controls the C–C bond formations, impacting the possibility of finding atoms at certain distances along the length of nanotubes.^5,26^ From figure panels, it seems that the number density of (8,4) chiral SWCNTs with a small *D* (=10.106 Å) is significantly higher as compared to (8,8) armchair (*D* = 13.232 Å) and (12,0) metallic zigzag (*D* = 11.460 Å) SWCNTs having large diameters. The greater ρ_*N*_ in (8,4) SWCNTs is a significance of its structure, which influences cross-sectional area, atomic arrangements, and bond energies and lengths, together leading to denser carbon atoms packing within SWCNTs. These types of such exclusive arrangements of bonds and angles can be observed in the higher ρ_*N*_, as shown in Figure 3d. This number density without strain clearly describes the atomic arrangements and angles within SWCNTs. This may also be correlated to Young’s Modulus of the SWCNTs (in forthcoming Figure 9).

**Figure 4:**
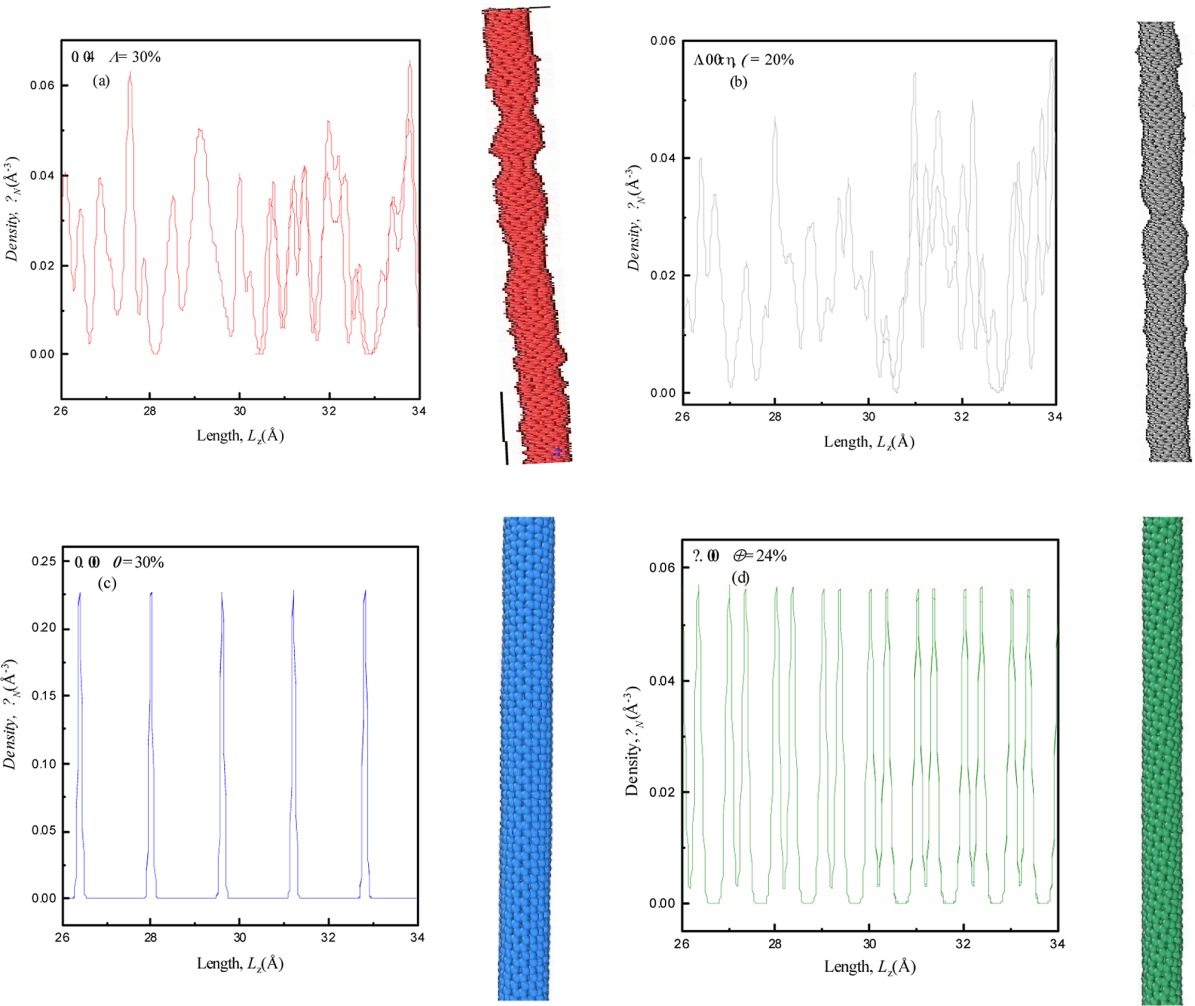
Density profiles obtained using the EMD simulation method along with corresponding structural visualizations (right side) at particular buckling strains (%) for (a) semiconducting zigzag (8,0), (b) metallic zigzag (12,0), (c) armchair (8,8) and (d) chiral (8,4) SWCNTs at *T* (K) = 300 K.

**Figure 5:**
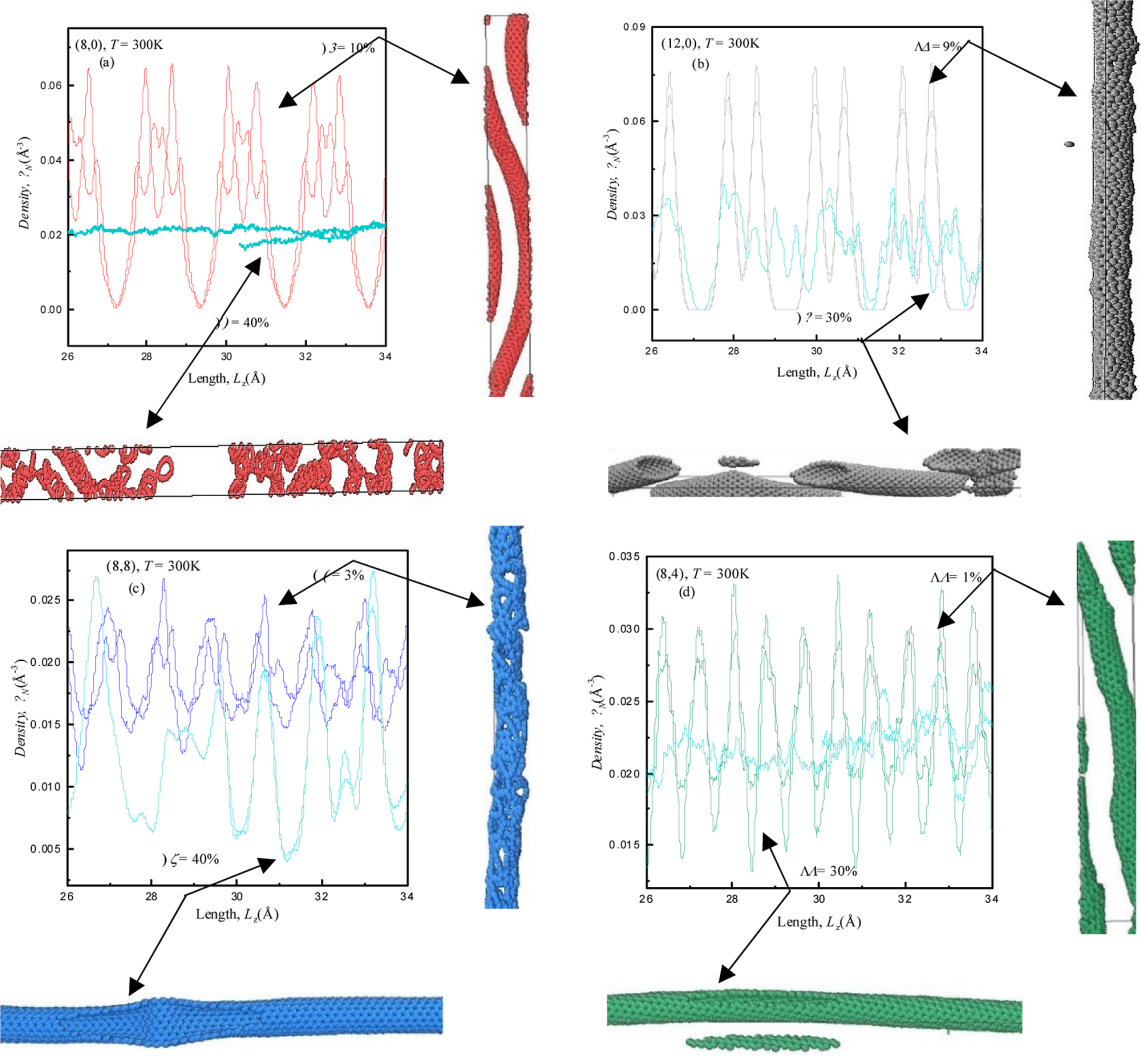
Density profiles obtained using the EMD simulation method along with corresponding structural visualizations (right vertical-compressive, bottom horizontal-tensile) at particular buckling strains (%) for (a) semiconducting zigzag (8,0), (b) metallic zigzag (12,0), (c) armchair (8,8), and (d) chiral (8,4) SWCNTs at *T* (K) = 300 K.

**Figure 6:**
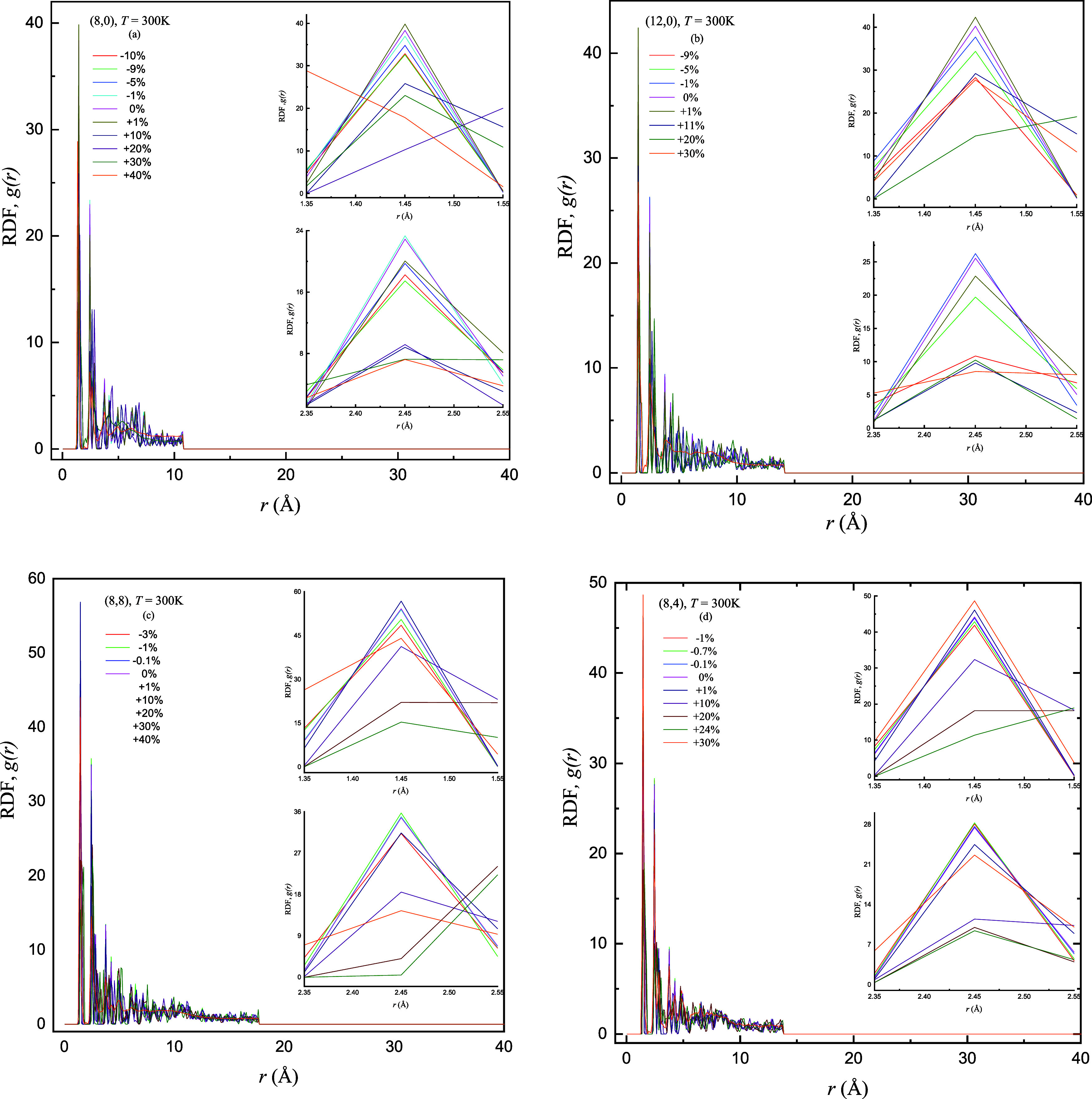
Comparison of obtained results of RDF *g*(*r*) as a function of interatomic distance *r* (Å) of the (a) semiconducting zigzag (8,0), (b) metallic zigzag (12,0), (c) armchair (8,8), and (d) chiral (8,4) SWCNTs by applying sequence of compressive −γ (%) and tensile +γ (%) strains at *T* (K) = 300 K. The inset figures display the prominent heights of first and second peaks of RDF for varying ±γ (%).

**Figure 7:**
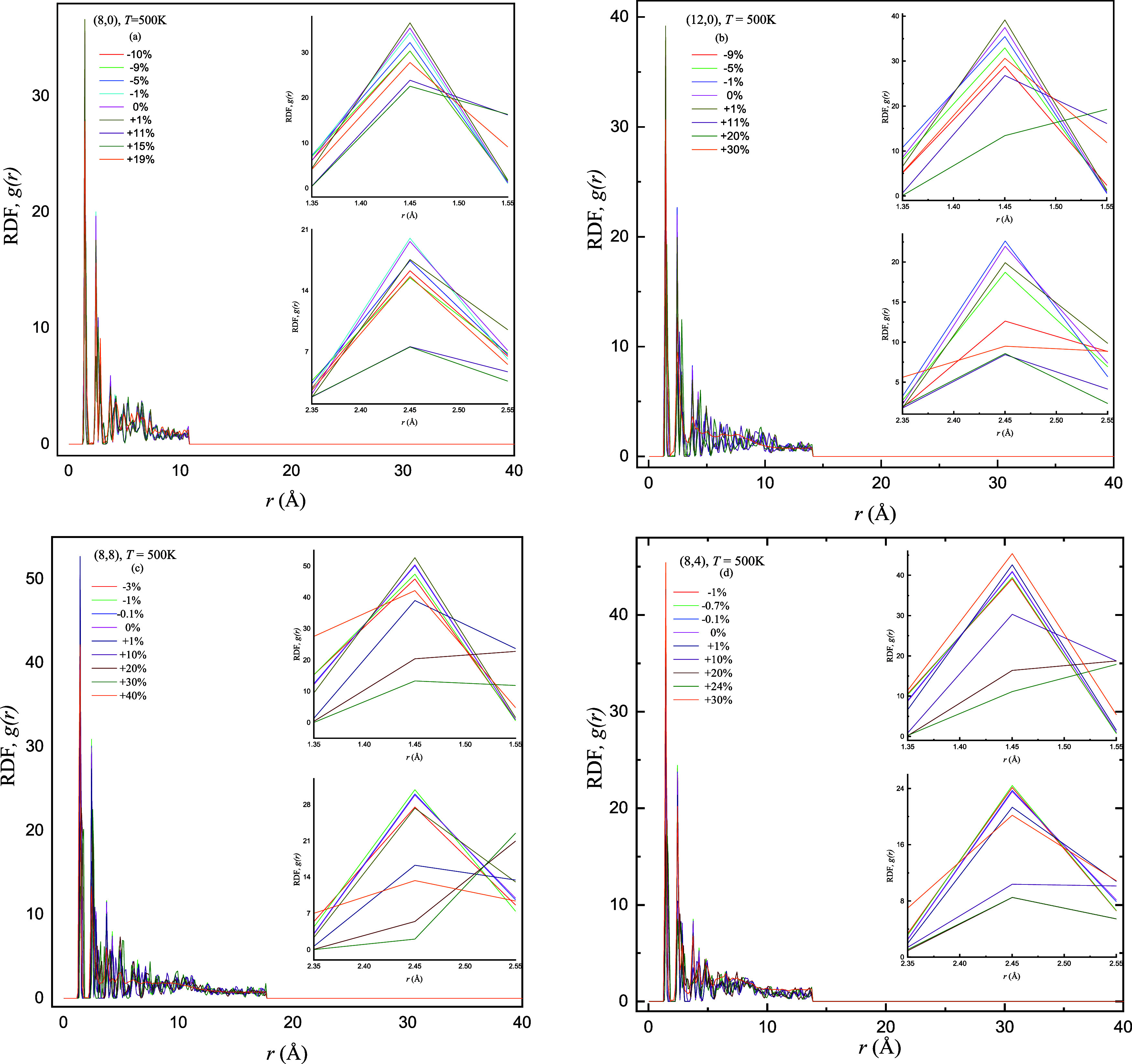
Comparison of obtained results of RDF *g*(*r*) as a function of interatomic distance *r* (Å) of the (a) semiconducting zigzag (8,0), (b) metallic zigzag (12,0), (c) armchair (8,8), and (d) chiral (8,4) SWCNTs by applying sequence of compressive −γ (%) and tensile +γ (%) strains at *T* = 500 K. The inset figures display the prominent heights of first and second peaks of RDF for varying ±γ (%).

**Figure 8:**
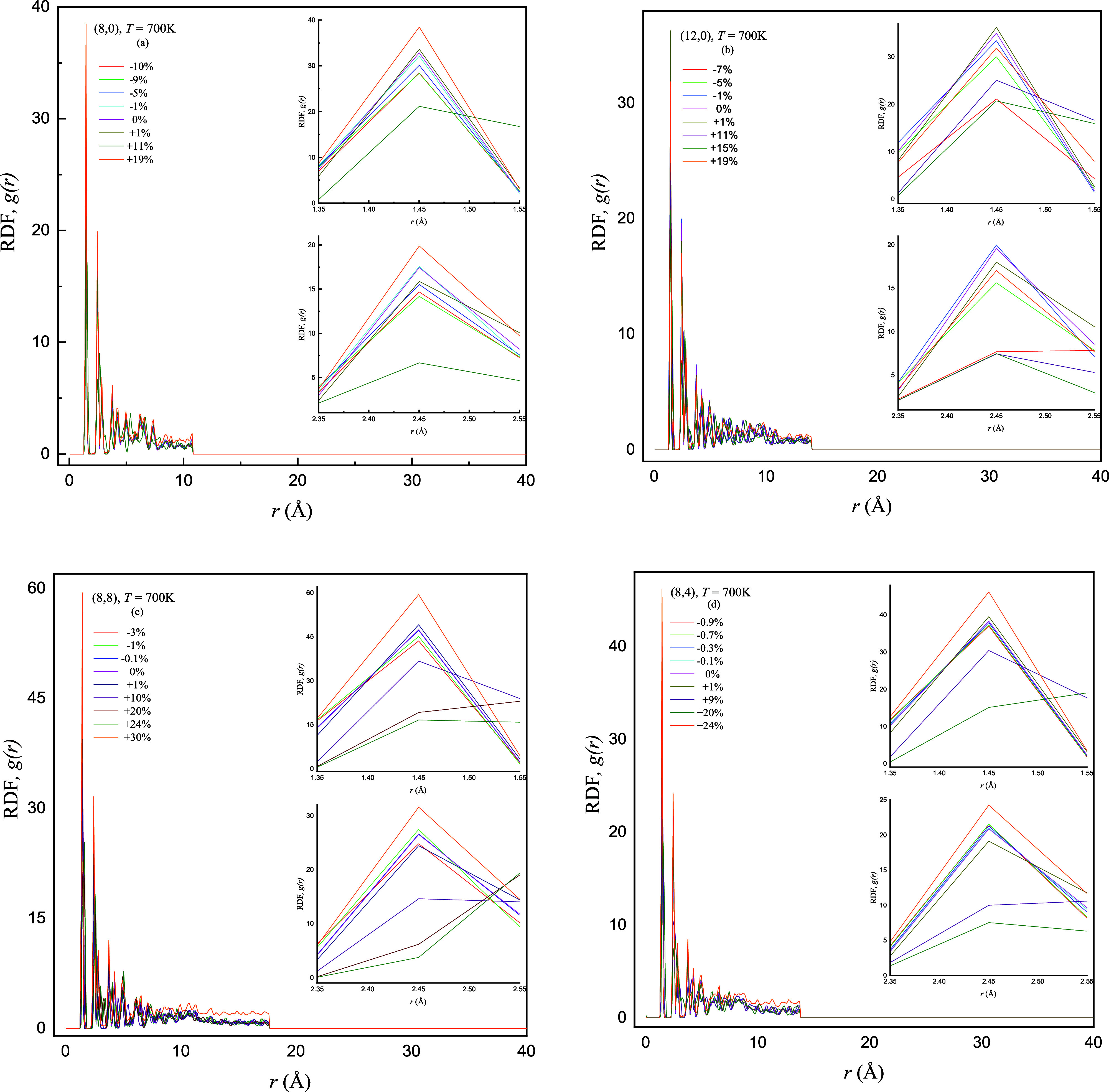
Comparison of obtained results of RDF *g*(*r*) as a function of interatomic distance *r* (Å) of the (a) semiconducting zigzag (8,0), (b) metallic zigzag (12,0), (c) armchair (8,8), and (d) chiral (8,4) SWCNTs by applying sequence of compressive −γ (%) and tensile +γ (%) strains at *T* = 700 K. The inset figures display the prominent heights of first and second peaks of RDF for varying ±γ (%).

**Figure 9:**
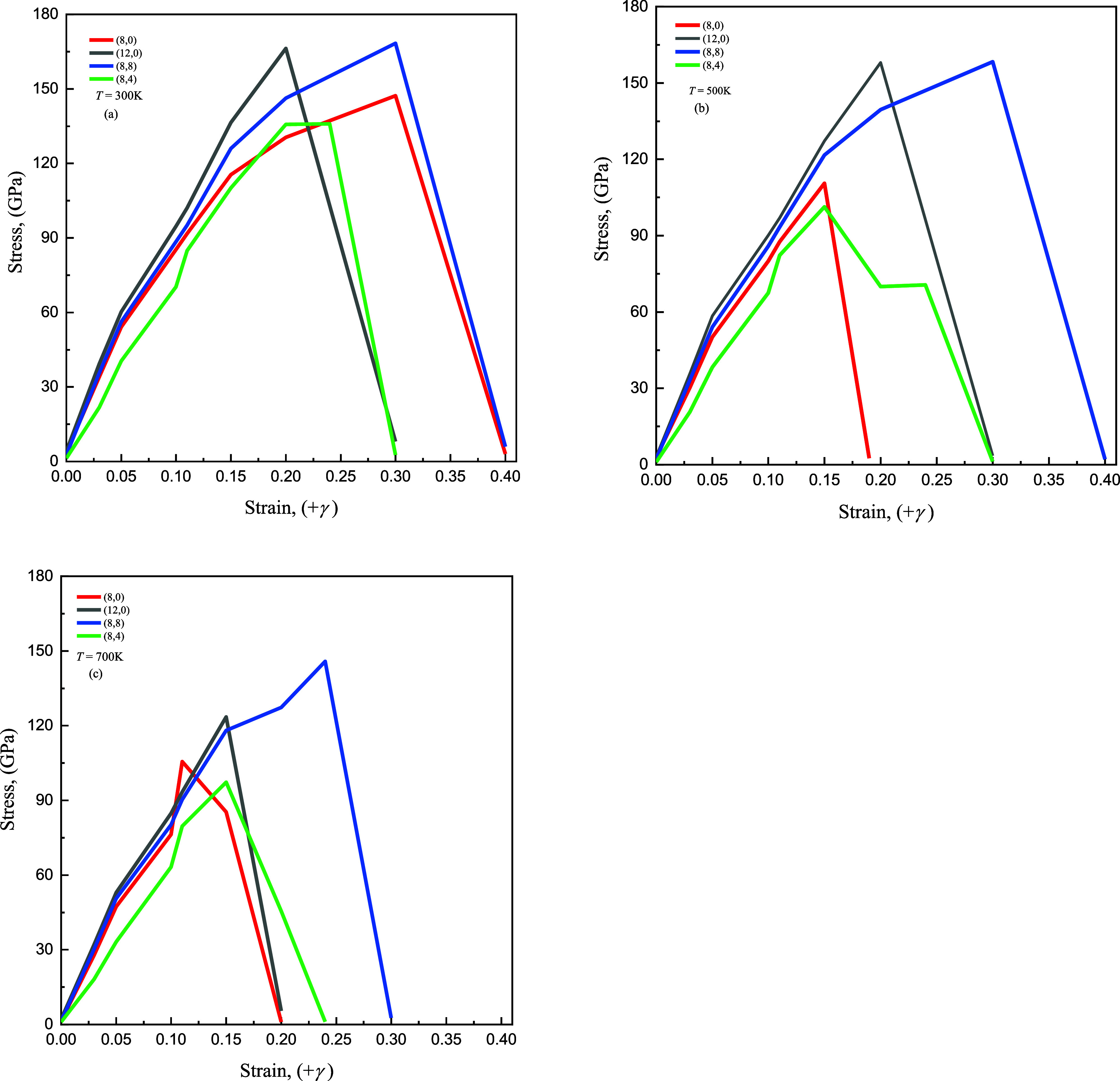
UTS test, stress as a function of strain for four configurations of SWCNTs at (a) *T* (K) = 300 K, (b) *T* (K) = 500 K, and (c) *T* (K) = 700 K.

Figure 4 represents the significant buckling shown through the density profile and visualizations on the right side of the respective SWCNTs in panels (a)–(d) at *T* (K) = 300 K. It can be clearly seen that (8,0) and (12,0) SWCNTs experience more buckling at +γ = 30 and 20%, respectively, in response to an applied strain. The density profiles and visualization through snapshots of zigzag SWCNTs reveal more prominent structural changes, involving bond elongation, leading to buckling, as shown in Figure 4a,b. Chirality and diameter influence on the strain threshold when structure changes and it becomes more prominent to explain how (8,0) requires a higher strain to buckle than (12,0). This characteristic is consistent with what will be detailed in our forthcoming RDF graphs [see Figure 6a,b], where the very last peak heights correspond to the buckle states. In contrast, (8,8) and (8,4) SWCNTs mostly experience stretching of carbon atoms due to tensile strains with less pronounced buckling at +γ = 30 and 24% as shown in Figure 4c,d, respectively. Density profiles and visualizations of the respective configurations demonstrate that the obtained behaviors exhibit a decrease in peak numbers corresponding to C–C bonding. This is due to the bond orientations and adjustment of interatomic distances, which result in reduction of buckling.^29^ Moreover, an important observation is noted here: the fall of peak heights as we have moved to such higher +γ (%) values. As will be explained in our forthcoming RDF graphs, the fall in peak heights is more obvious for zigzag SWCNTs as compared to armchair and chiral ones [Figure 6a–d]. The stretching in the structure of nanotubes is mainly due to tensile strains, and it decreases the distances among atoms in nanotubes.

Furthermore, concerning (8,0), (12,0), (8,8), and (8,4) SWCNTs at *T* (K) = 300 K, the deformation process under ±γ (%) is considered along with density profiles and respective deformed visualizations in right vertical [−γ (%)] and bottom horizontal [+γ (%)] sides (front views) of corresponding panels (a) to (d) of Figure 5. Density profile reveals the overall variations in peaks within a range of 0.0 ≤ peaks ≤0.07, highlighting differences in structural responses of SWCNTs, for −γ (%). It is observed that the (8,0) and (12,0) nanotubes deform at high compressive strains (−γ = 10 and 9%) as compared to (8,8) and (8,4) nanotubes which lead to deform at low compressive strains (−γ = 3 and 1%). Zigzag SWCNTs have a more densely packed arrangement of carbon atoms, resulting in stronger interatomic bonding compared to armchair configurations and chiral ones when subjected to −γ (%) strains.^30^

Visualizations through snapshot frames of (8,0) and (8,4) show more distorted nanotubes, as shown in Figure 5a,d because of the small diameter of both nanotubes, whereas (12,0) and (8,8) display less deformed nanotubes, as shown in Figure 5b,c, when exposed to −γ (%) strains. A combined effect of chirality and diameter was confirmed through visualization of each configuration shown adjacent to density profiles and also the peaks in forthcoming RDF graphs [see Figure 6a–d] for −γ (%).

Now dealing with tensile strains +γ (%) employed in different configurations of SWCNTs, panels (a) and (c) of Figure 5, it is examined that the deformation process starts at high +γ = 40% for (8,0) and (8,8), whereas (12,0) and (8,4) SWCNTs deform at +γ = 30% as shown in Figure 5b,d. Flexibility and bond orientation and elongation to strains can provide higher mechanical strength to semiconducting zigzag (8,0) and armchair (8,8) SWCNTs to withstand large tensile strains as compared to the large diameter metallic zigzag (12,0) and chiral (8,4) SWCNTs.^31^

The density profile indicates that a high fluctuation is observed for zigzag SWCNTs [see Figure 5a,b] as compared to the other two configurations of SWCNTs. Structural visualizations of (8,0) and (8,8) SWCNTs also confirm more destructive (maximum deformation) patterns as has been observed in our preceding discussion [see Figure 4a,b] of more buckled (maximum buckling) nanotubes with +γ (%). This corresponds to more buckled nanotubes with a higher degree of buckling +γ (%). The negligible deformed arrangement is observed for (8,8) and (8,4) SWCNTs as we have already discussed [see Figure 4c,d] the less buckling arrangement due to tensile strains +γ (%). In addition, our forthcoming RDF graphs [Figure 6a–d] discuss the confirmation of buckling and deformation patterns through peak heights. It is concluded from Figures 3–5 that (8,0) and (8,8) SWCNTs are later deformed for higher tensile strain +γ (=40%) as followed by (12,0) and (8,4) SWCNTs earlier deformed for tensile strain +γ (=30%) at *T* (K) = 300 K. These observations are connected to intrinsic structural characteristics, chirality, diameter, and temperature of the SWCNTs in the case of tensile strains pointing to the fact that zigzag followed by armchair can bear more strain and be useful for applications demanding robust and high-performance materials, strain sensors, nanomechanical devices, and nanocomposites.^32^ The density profiles and structural visualizations further confirm the varying responses of each configuration by modulating ±γ (%).^33^

### 3.3 Radial Distribution Function

To unveil complete structural information regarding complicated interconnection between *T* (K), *L* (Å), and chirality, the RDF *g*(*r*) graphs are plotted against varying ±γ (%). RDF analysis is employed to evaluate the structural stability of strained ±γ (%) and unstrained SWCNTs at different *T* (K) = 300, 500, and 700 K. The insets of the respective graphs highlight the first and second peaks of *g*(*r*) at varying ±γ (%) in depth. These insights arise from a careful exploration of varying ±γ (%) in which buckling and deformation limits are observed and these limits may strongly depend on the chirality of SWCNTs despite the same *L* (Å). The RDF peaks provide a better vision of the structural characteristics and atomic arrangements of the SWCNTs. Figures 6–8 provide information regarding the compressive and tensile deformation limits [threshold values of ±γ (%)] during analyzing the *g*(*r*) plots of (8,0), (12,0), (8,8), and (8,4) SWCNTs. The reduction in peak heights before the deformation limit is reached is credited to the adjustments in atomic spacing and elongation (and/or compression) of carbon bonds^34^ as SWCNTs undergo varying strains. It is interesting to mention here that distinct buckling and deformation behaviors are observed for each nanotube configuration with the same *T* (K) and *L* (Å).

Figures 6–8 display the increasing sequence of ±γ (%) applied on the zigzag (semiconducting and metallic), armchair, and chiral SWCNTs at *T* (K) = 300, 500, and 700 K, respectively, labeled as (a), (b), (c), and (d) in respective panels. The RDF patterns demonstrate the structural stability of unstrained and strained SWCNTs, and they focus on the variation of tuned peak heights that show the buckling and deformation behaviors within different configurations of nanotubes. Four panels of each figure show the computationally traceable buckling and deformation limits found roughly between 0 ≤ ±γ (%) ≤ 40 for the EMD algorithm, which depends on the nanotube parameters (*T*, *L*, chirality, and diameter). Particularly, the (8,8) SWCNTs consistently reflect the highest peak followed by the (8,4), (12,0), and (8,0) nanotubes, and this trend is credited to the distinctive angles and bond orientations of each corresponding SWCNTs. The unique chirality and geometric configuration of (8,8) SWCNTs create conditions where the possibility of finding atoms from the reference point is maximum; consequently, the highest peak in the RDF graph is observed. Structural impact on peak heights emphasizes the major influence of chirality and geometry on *g*(*r*) of SWCNTs with varying *T* (K). As SWCNTs share varying diameters and lengths (due to strain), they critically influence the probabilities of peak occurrences.

The decrement in peak heights with increasing +γ (%) is more pronounced compared to an applied −γ (%). It is already examined that the (8,0) and (8,8) SWCNTs buckle at +γ = 30% followed by the (8,4) at +γ = 24% and (12,0) at +γ = 20% and *T* (K) = 300 K. We advance one possible reason for the reduction of peak heights that the external force due to an applied +γ (%) causes the nanotube to stretch, leading to bond elongation among carbon atoms and it results in a gradual increase of interatomic distances and so decrease in peak heights.^5^ It is mentioned here that the CNT structure may become unstable at a certain and/or maximum buckling point and soon after the nanotube structure deforms, which may lead to a very sharp and prominent increase/decrease in the peak height. This sudden and/or abrupt shift in peak height causes a structural transition beyond which the C–C bond rupturing may happen in CNTs and the rapid increase in peak heights indicates the prominent atomic rearrangement, leading to possible bond deformation processes in CNT structure.^26^

It is noted through analyzing the first and second peaks that the maximum peak height is observed for (8,8), then (8,4) followed by the (12,0) and (8,0) SWCNTs and the first peak heights of all configurations are close to each other as compared to the second peak heights. It is evident from panels of Figure 6a,c that the (8,0) and (8,8) SWCNTs are more stable and can withstand a broad range of tensile strains +γ (=1 to 30%) before breaking at low *T* (K)= (300 K). The deformation process is started in (8,0) and (8,8) SWCNTs at +γ = 40%, whereas it started in (12,0) and (8,4) SWCNTs at comparatively less +γ = 30%. At deformation points (+γ = 30 and 40%), an abrupt shift and/or increase in peak height is noted, reflecting the SWCNT structure breakdown and it may be seen in preceding Figure 5 (horizontal views). Regardless of the same configuration, (8,0) can withstand higher tensile strains because of its small diameter as compared to (12,0). So, the (8,0) and (8,8) SWCNTs have stronger and more stable interatomic bonding, making them susceptible to break at higher tensile strains.

In the case of the deformation process, diameter and configuration can play a significant role in the decrement and/or increment of peak heights of RDF. The peak heights of zigzag and armchair SWCNTs are definitely decreased at deformed strain points as compared to peak heights without strains [+γ (%) = 0]; however, the peak height of chiral SWCNTs is slightly increased at *T* (K) = 300 and 500 K, as shown in Figures 6 and 7. We may advance one possible reason for the reduction of RDF peak heights that can cause the extension of the lattice structure of nanotubes with an applied +γ (%) and this extending nanotube is more pronounced at low-intermediate *T* (K). Normally, with an applied +γ (%) to the nanotubes, the distance among atoms *r* (Å) increases, leading to a decrease in the RDF peak heights. At further high *T* (K) = 700 K, a converse effect is observed at breaking +γ (%) strains that the peak height is slightly increased in contrast to peak height at +γ (%) = 0 for (8,0), (8,8), and (8,4) SWCNTs. It is observed that the first and second RDF peak heights decrease at +γ (%) = 0 and definitely increase at breaking +γ (%) with an increase in *T* (K) and the difference of RDF peak heights is comparatively more pronounced for (8,0) and (8,8) SWCNTs. However, at high *T* (K) (=700 K), it causes the thermal fluctuations among atoms that can lead to fragile nanotube structures and it subjects the nanotube to deform at low value of +γ (%) strains.^14,35^ It may cause abrupt changes (increase and/or decrease) in RDF peak heights due to the nanotube lattice structure deformed at earlier +γ (%) strains and high *T* (K), as shown in Figure 8.

It is interesting to note here that the buckling and deformation points regarding +γ (%) strains remain the same for (12,0), (8,8), and (8,4) SWCNTs with increasing *T* (K) = 300 to 500 K, but the (8,0) nanotube buckles at +γ = 15% and breaks at +γ = 19%. Moreover, the buckling and deformation values decrease as *T* (K) increases for all configurations. The strong thermal vibrations at a high temperature (700 K) may lead to buckle and break at earlier +γ (%) strains and these are observed at more earlier +γ = 11% in (8,0) which may be due to small diameter. Initially, when *T* (K) is low, thermal vibrations are only prominent in nanotubes with small diameters (chiral SWCNTs), but when *T* (K) is continuously increased, then it becomes enough to distort the peak heights completely. The (12,0) SWCNTs are excepted and it may be due to its larger diameter as compared to other SWCNTs. The system temperature *T* (K), the strained values ±γ (%), chirality, nanotube length *L* (Å) and diameter *d* (Å), simulation time step (d*t*), thermal effects, and simulation proceed length (total run time) are varied to estimate how these parameters can influence the earlier buckling and deformation of SWCNTs and nanotubes become very useful even when it buckled at high temperature.^36^

It is evident from the four panels of Figures 6–8 that the peak heights decrease with increasing −γ (%) strains and peak heights are sharper as compared to peak heights with +γ (%) strains. We propose here one possible reason for this reduction of peak heights that may happen from the compression in the lattice structure of SWCNTs and it explains the decrease in RDF peak heights by applying −γ (%) strains. The semiconducting zigzag SWCNTs have a maximum value of breaking/buckling compressive −γ (%) strains, and the lowest value corresponds to chiral SWCNTs. The sharp sequence and difference in peak heights are more pronounced for −γ (%) strains as compared to the sequence and difference in peak heights for the +γ (%) strains. However, the range of compressive −γ (%) strains is more than two (and/or three) times less than the range of tensile +γ (%) strains, depending on nanotube chirality and system *T* (K). It is noted that the drop in first and second peak heights is relatively less with employing successive −γ (%) = 1 to 10% strains, and peak heights slightly decrease and the difference in peaks becomes smaller with increasing *T* (K). Furthermore, the peak heights (first and second) by applying −γ (%) strains are significantly higher and less broad as compared to peak heights with +γ (%) strains. In four panels of Figures 6–8, as the −γ (%) strain increases at varying *T* (K), the coordination number of nearest neighbor atoms increases with decreasing atomic separation *r* (Å), leading to high sharp RDF peaks in nanotubes. The buckling and breaking points of zigzag, armchair, and chiral SWCNTs remain the same with increasing *T* (K); however, the breaking point slightly decreases for (12,0) and (8,4) SWCNTs at high *T* (K) = 700 K. At high *T* (K), the thermal vibrations among atoms of nanotubes are increased and it can cause to increase in the average bond length in SWCNTs,^13^ reducing the effect of compression on nanotubes. Therefore, it is concluded that the bearing capacity of compressive strains is large for zigzag SWCNTs as compared to other configurations (armchair and chiral), irrespective of varying *T* (K). The bond (C–C) compression tends to be very strong and it leads to fast buckling but slow decrement in RDF peak heights.

The observed drifts in the RDF peaks are the consequence of the complicated interplay between bond compression, elongation, and ultimate structural changes due to applied ±γ (%) strains. Unique arrangements and orientations of bonds in each configuration of SWCNTs dictate the reaction to compressive and tensile strains, leading to deviations in peak heights and deformation behaviors. Finally, it is summarized that the buckling phenomenon is more prominent in the case of tensile strains depending on *T* (K) and that the SWCNTs can bear more tensile strains as compared to compressive strains, ensuring their reliability and functionality. Considering these patterns can deliver valuable information for adapting SWCNT properties in various applications, from materials engineering to nanoscale mechanics; therefore, materials can be tailored that can display superior performance when subjected to strain/stress conditions. Flexible strain/pressure sensors are very crucial in wearable electronics because of their respective working mechanism.^37^

### 3.4 Stress–Strain Analyses

Three panels of the plot are shown to elaborate the analysis of MPs of the simulated SWCNTs under varying uniaxial tensile strains at three systems, *T* (K), as displayed in Figure 9. Simulations for four different configurations of SWCNTs (semiconducting and metallic zigzag, armchair, and chiral) to consider the reliability and precision of the MPs were performed and drawn in panels (a), (b), and (c) of Figure 9. In the case of *T* (K) = 300 K [and/or *T* (K) = 500 K and *T* (K) = 700 K], we have compared different calculations corresponding to four configurations of SWCNTs (a total of nine simulations data sets for three temperatures) until their deformation limits are reached. Stress–strain curves provide the analyses and variations in ultimate tensile strength UTS (GPa), ultimate strain (US), and Young’s modulus *Y* (Pa) influenced by the chirality, diameter, and *T* (K). It is obvious from the stress–strain graph that the stress increases initially with an increase in +γ strain, elaborating linear elastic behavior and also confirming earlier findings.^9,11,19,25^ It is observed from three panels of Figure 9 that the value of stress increases with increasing +γ strains for (8,0), (12,0), (8,8), and (8,4) SWCNTs and reaches its maximum buckling points. A significant behavior is observed at particular strains (yield strength) where the stress level continues to increase as mentioned in previous results.^11,25^ This behavior happens to be normal in most SWCNTs representing essentially possible improved ductility/buckling phenomena, as it may be revealed through the variation between the maximum Young’s modulus, UTS, and probably yield strength. It is observed that the maximum of stress is noted near around ∼168.32 GPa for the configuration of armchair (8,8) and the lowest value of stress near ∼ 135.734 GPa for chiral (8,4), while (8,0) and (12,0) zigzag configurations have intermediate stress values of ∼ 147.202 and ∼166.341 GPa, respectively, at *T* (K) = 300 K. These highest stress levels are observed corresponding to each configuration of SWCNTs at particular strains where the SWCNTs are maximum buckled. Likewise, the pattern of maximum, minimum, and intermediate values of stress can be seen corresponding to higher *T* (K) = 500 K (700 K), respectively, as ∼158.33 GPa (∼145.774 GPa) for armchair, ∼ 101.272 GPa (∼97.312 GPa) for chiral, ∼ 110.54 GPa (∼105.559 GPa) for semiconducting zigzag, and ∼157.981 GPa (∼123.558 GPa) for metallic zigzag SWCNTs. The stress level decreases with an increase in *T* (K) and the stress level (highest) corresponding to armchair (8,8) SWCNTs is very close to the stress level (second highest) of metallic zigzag (12,0) SWCNTs, as expected. In the case of *T* (K) = 300 K, it is established that the yield strength is measured as ∼42 GPa (chiral), ∼ 60 GPa (metallic zigzag), ∼56 GPa (semiconducting zigzag), and ∼58 GPa (armchair). Moreover, the yield strength is calculated as ∼38 GPa (chiral), ∼ 58 GPa (metallic zigzag), ∼ 50 GPa (semiconducting zigzag), and ∼56 GPa (armchair) for the case of *T* (K) = 500 K and ∼20 GPa (chiral), ∼ 54 GPa (armchair), ∼ 46 GPa (semiconducting zigzag), and ∼48 GPa (armchair) for the case of *T* (K) = 700 K. It is interesting to note here that the yield strength of (8,4) configuration decreases but the (8,8) configuration increases with increasing *T* (K); however, high yield strengths are examined for the armchair (8,8) configuration that are also very close to strengths in semiconducting (12,0) SWCNT configuration for all three *T* (K), as expected.

After the linear region and reaching the buckling point, the stress–strain graph decreases sharply, illustrating the nanotube plastic deformation with further continuous fast drop in the stress level where the UTS is the maximum stress that SWCNTs may have in the plastic deformation region. Three panels of Figure 9 show that the UTS values are calculated between 136 and 97 GPa (chiral), 168 and 146 GPa (armchair), 166 and 124 GPa (metallic zigzag), and 147 to 106 GPa (semiconducting zigzag) for +γ strains varying between 0.11 and 0.4, depending on increasing *T* (K) = 300, 500, and 700 K. It is observed that the UTS decreases with increasing *T* (K) and the highest value of UTS is noted for armchair SWCNTs as compared to other configurations. However, the minimum change in UTS (22 GPa) is observed for armchairs and the maximum change is measured for both zigzag (41 and 42 GPa) SWCNTs with an increase in *T* (K). From the stress–strain plot, it is noted that a fast drop in maximum stress showing in the plastic region is observed, as deformation is expected.^9^ Armchair and zigzag SWCNTs have highest UTS than chiral, and this maximum stress of nanotubes can withstand before their maximum bonds rupture at different *T* (K). These high UTS are primarily due to the unique arrangement of C–C bonds in armchair and zigzag configurations of SWCNTs. At high *T* (K) (=500 K and/or 700 K), the range of UTS starts to decrease due to high thermal vibrations which makes the nanotube structure more susceptible to deformation as in panels of Figure 9b,c. It should be mentioned here that the (8,0) and (8,8) SWCNTs bear more strain (=0.3) in contrast to the other two configurations. UTS falls in the range of 101 GPa (111 GPa) for ultimate strains of 0.15 (0.24) indicating that (8,0) [(8,4)] SWCNTs can still experience elongation before failure. It is examined that (8,0) displays more stress (111 GPa) regardless of the low ultimate strain (0.15) as compared to (8,4) which bears more stress (101 GPa) whereas ultimate strain is (0.24). The third panel of Figure 9c provides the lowest values of UTS at high *T* (K) = 700 K and it predicts that high *T* (K) reduces the ability to bear ultimate stress more sharply as compared to strain. UTS range falls from 146 to 97 GPa with an ultimate strain of 0.11 to 0.24, signifying the reduction in SWCNTs’ mechanical strength to resist tensile strain. SWCNTs drive strength directly from armchair and zigzag structures that do not have any dislocations or defects that limit the strength of chiral.

Moreover, Young’s modulus is found to be in the range of 0.50–0.83 TPa, at *T* (K) = 300 K and it overall decreases as *T* (K) increases. Our outcomes are in satisfactory agreement with earlier known experimental investigations^21^ and MD simulations^9,18,19^ and illustrate that the current data using EMD simulations and earlier methods have comparable efficiency, both providing close values for Young’s modulus. Young’s moduli change growing order with increased diameter and strains for four configurations. The comparatively high Young’s modulus value along with high UTS, ultimate strain, and yield strength, in the case of armchair and metallic zigzag SWCNTs, predict that the particular interface region contributes a significant task in the nanotubes. The drawback of low plasticity has been improved with the creation of two-phase microstructures consisting of a ductile reinforcement material in strained nanotubes.

Furthermore, a distinct series of eight further simulations is performed to the influence of varying *T* (K) = 500 and 700 K on the stress–strain analysis for four configurations of SWCNTs which are shown in Figure 9b,c. It indicates that the value of stress is high for (8,8) and (12,0) and stress values of these configurations are nearly close to each other at both *T* (K) = (500 and 700 K); however, the stress level is higher for all configurations at *T* (K) = 300 K. It is calculated as that the value of UTS is between 0.42 and 0.80 and between 0.49 and 0.96 TPa for *T* (K) = 500 and 700 K, respectively. An increment in *T* (K) causes the thermal vibrations that lead to buckle, as shown in preceding Figures 5–8. This possible expansion may reduce C–C interatomic interactions, resulting in stiffness degradation and hence reduce Young’s modulus.^18,19^ It can be interpreted that the overall UTS for (12,0) and (8,8) configurations are definitely higher than that of (8,0) and (8,4) SWCNTs, elaborating that the plasticity limit for (12,0) and (8,8) is higher than (8,0) and (8,4) at varying 300–700 K. However, at *T* (K) = 700 K, the UTS value of (8,0) is higher than that of the rest of configurations and the limit of plasticity of (8,0) is higher than (8,8) and (8,4) at high *T* (K) = (500 and 700 K). The last two panels of Figure 9 suggest that the flow of stress decreases with an increase in *T* (K) and it can be due to fast diffusion of free density at high *T* (K). It can be predicted that this fast diffusion of free density is less for (8,0) and higher for (8,4) as *T* (K) increases.

It is summarized from plots that the reported simulations can precisely suggest the structural study and MPs of SWCNTs at varying strains ±γ (%). The comparison of expected yield strength, ultimate strain, UTS, and Young’s modulus is that these MPs are maximum for metallic zigzag (12,0) and armchair (8,8) and minimum for chiral (8,4) SWCNTs. It is suggested that these MPs [yield strength, ultimate +γ, UTS, and *Y* (Pa)] and structural analyses are tuned at varying *T* (K) and ±γ (%). It is obvious that the MPs have intermediate values for semiconducting zigzag (8,0) and it may be due to its short diameter but high buckling and breaking strains. It seems that (8,0)/(8,8) has maximum plastic strain and (8,4)/(12,0) has minimum plastic strain. It is predicted that the ductility is generated to decrease with increasing buckling/breaking strains for (8,4)/(12,0) with intermediate diameters. It is suggested that the (8,0)/(8,8) SWCNTs illustrate more ductility along with higher buckling/breaking strains as compared to (8,4) and (12,0) SWCNTs, and consequently, the configurations with intermediate diameters fracture first, whereas the configurations with small-large diameters fail in the end. It is recommended that the brittleness of the configurations may be improved with increasing diameters.

## 4 Conclusions

Structural analyses and MPs of zigzag-armchair-chiral-based SWCNTs are investigated through MD simulations and the effects of varying strains ±γ (%) and system *T* (K) of four SWCNTs are studied. The varying ±γ (%) buckling and deformation of (8,0), (12,0), (8,8), and (8,4) SWCNTs have displayed that the buckling and deformation processes of nanotubes are diverse from those without strains due to the anisotropic nature of the CNT bonding, and the corresponding geometries show distinctive behavior. Convergence analyses help investigate the SWCNTs’ responses toward ±γ (%) and *T* (K) that provide visions into their load-bearing capacity, stiffness, buckling, and flexibility. It is demonstrated that the buckling process is more pronounced for tensile strains depending on *T* (K) and configurations of SWCNTs may be near more tensile strains as they contract to compressive strains, confirming the reliability and functionality. The obtained results show that the zigzag-armchair-chiral-based SWCNTs provide uniaxial deformation under tensile trials for varying *T* (K). It is shown that the zigzag-armchair-chiral-based SWCNTs have limited deformation as contact to brittle fracture during ultimate +γ deformation. The MPs such as YS (GPa), UTS (GPa), and *Y* (TPa) of four SWCNT configurations decrease with increasing *T* (K) and MPs of (12,0) and (8,8) SWCNTs are nearly close to each other and comparatively higher as compared to (8,0) and (8,4) SWCNTs. The moderately high diameter increases the yield strength, UTS, and Young’s modulus of the metallic zigzag and armchair SWCNT configurations and probably decreases the short-range ordering. The (8,8) SWCNTs have higher values of UTS indicating the nanotube/nonmetallic structures that openly force strength in SWCNTs and are not responsible for any defects (or dislocations) that hurdle strength of C–C structures, at room *T* (K). On the other hand, the (8,0) SWCNT has a high value of Young’s modulus and highlights that it is stiffer in contrast to (12,0), (8,8), and (8,4) SWCNTs, at high *T* (K) = 700 K. Interestingly, the plasticity boundary is improved for (12,0) and (8,8) than the other two SWCNTs at room *T* (K). MPs of SWCNTs can be enhanced by considering the exact approximation of tensile strains where buckling or deformation can be useful.
